# Immune Microenvironment Features and Dynamics in Hodgkin Lymphoma

**DOI:** 10.3390/cancers13143634

**Published:** 2021-07-20

**Authors:** Clara Bertuzzi, Elena Sabattini, Claudio Agostinelli

**Affiliations:** 1 Haematopathology Unit, IRCCS Azienda Ospedaliero-Universitaria di Bologna, 40138 Bologna, Italy; elena.sabattini@aosp.bo.it (E.S.); claudio.agostinelli@unibo.it (C.A.); 2 Department of Experimental, Diagnostic and Specialty Medicine, University of Bologna, 40138 Bologna, Italy

**Keywords:** Hodgkin lymphoma, microenvironment, immune escape, immune surveillance

## Abstract

**Simple Summary:**

As happens in all neoplasms, the many reciprocal interactions taking place between neoplastic cells and the other reactive cells impact the course of the disease. Hodgkin Lymphoma is an haematologic malignancy where most of the pathological tissue is indeed composed by reactive cells and few neoplastic cells. Consequently, it represents an interesting subject for the description of the neoplastic and non-neoplastic cells interaction. In this review we report and discuss the more recent findings of microenvironmental studies about this disease.

**Abstract:**

Classical Hodgkin’s lymphoma (cHL) accounts for 10% of all lymphoma diagnosis. The peculiar feature of the disease is the presence of large multinucleated Reed–Sternberg and mononuclear Hodgkin cells interspersed with a reactive microenvironment (ME). Due to the production of a large number of cytokines, Hodgkin cells (HCs) and Hodgkin Reed–Sternberg cells (HRSCs) attract and favour the expansion of different immune cell populations, modifying their functional status in order to receive prosurvival stimuli and to turn off the antitumour immune response. To this purpose HRSCs shape a biological niche by organizing the spatial distribution of cells in the ME. This review will highlight the contribution of the ME in the pathogenesis and prognosis of cHL and its role as a possible therapeutic target.

## 1. Introduction 

Starting from its description in 1832 [1]. Hodgkin lymphoma (HL) captured the attention of scientists that clarified the nature of the disease throughout time. Classical Hodgkin’s lymphoma (cHL) accounts for 10% of all lymphoma diagnosis. The peculiar feature of the disease is the presence of large multinucleated Reed–Sternberg and mononuclear Hodgkin cells interspersed with a reactive microenvironment (ME) [1]. The latter can represent more than 95% of the entire tumoral mass and consists of T and B lymphocytes, neutrophils, eosinophils, macrophages, plasma cells, mast cells, fibroblasts and vessels [2]. The type and the number of these populations in the inflammatory background determine the distinction of cHL in histological subtypes: nodular sclerosis, mixed cellularity, lymphocyte depleted and lymphocyte rich [2]. The origin of the Hodgkin RS cells (HRSCs) has long been unknown, until molecular studies conducted by the microdissection of tissue sections and PCR analysis for rearranged immunoglobulin (IG) genes have shown that most HRSC and H cells (HCs) derived from peripheral B-lymphocytes, although a clonal rearrangement of the T-cell receptor genes was occasionally demonstrated (2% of cases) [2]. The immunophenotype and the genetic features of HC and HRSC are identical in these subtypes, but their clinical features are different. HRSC and HC express the CD30 and CD15 molecule in more than 98% and in about 75% of cases respectively [2]. Despite their mature B cells derivation, HRSC and HC are usually negative for the canonical B cells markers [1]. This feature was related to the downregulation, by epigenetic processes, of B-cell-specific transcription factors including PU.1, Oct.2 and its coactivator BOB.1 [3]. The B-cell origin of HRSC is demonstrated only by the expression of the transcription factor PAX5 [4].

Genetic instability is a characteristic of cHL that exhibits numerical chromosome aberrations usually in the hyperploid side. Several studies demonstrated that the constitutive activation of NF-kappaB sustains proliferation and prevents apoptosis in HRSC and HC. Different mechanisms underlie this phenomenon: gain/amplifications of REL (in 30% of the cases) and BCL3 oncogenes, aberrant activation of the natural inhibitors of NF-kappaB as the I-kappaB family or A20, the constitutive activation of AP-1 and LMP-1 overexpression (in EBV+ cases) [5,6,7,8,9,10]. In about 90% of cases cHL shows dysregulation, resulting in increased activation, of the JAK-STAT pathway by genetic alterations in multiple genes, such as STAT6 (32% of cases) STAT3, STAT5B, JAK1, JAK2 and the negative regulator PTPN1 [11]. Additional lower frequency somatic mutations of GNA13, XPO1 and ITPKB genes have also been reported [11]. After standard chemotherapy regimens with ABVD (doxorubicin, bleomycin, vinblastine and dacarbazine) and radiotherapy when indicated, almost all patients with cHL have a remission; however, 30% of the advanced stages and 15% of early stages have a relapse after treatment [12]. Early relapse defines a drug resistant subgroup associated to poor prognosis throughout all subsequent treatments. The introduction of the highly targeted antibody–drug conjugate brentuximab vedotin (BV), which delivers the cytotoxic drug monomethyl auristatin E (MMAE) to CD30-positive cells, represented an important step forward in the treatment of relapsed and refractory disease and the addition of BV to an AVD scheme is effective in advanced disease cHL, particularly in patients with a contraindication for receiving bleomycin [13]. Furthermore, in refractory cHL the immunotherapy with immune checkpoint inhibitors pembrolizumab or nivolumab, reverting the block of PD1 signalling pathway, has reached outstanding results with high response rates and quite durable disease control [14,15]. However, their precise mechanisms have not been clarified as they target not only the neoplastic cells but also the ME and, therefore, further studies are needed to explain the microenvironmental dynamics and the interaction between the tumour and immune system. This review will highlight the contribution of the ME in the pathogenesis and prognosis of cHL. The ME configuration depends on several complex and entangled mechanisms through which HRSC shape their own biological niche: they first recruit the immune population, then functionally reprogram them to receive survival stimuli and to escape the immune surveillance. 

## 2. Recruitment and Constitution of the Microenvironment

As happens in all neoplasms, the many reciprocal interactions taking place between HRSC and the other reactive cells impact the course of the disease.

The HL ME is constituted by B and T lymphocytes, plasma cells, eosinophils, macrophages, mast cells myeloid suppressor derived cells and NK cells in a variable proportion.

**Figure 1:**
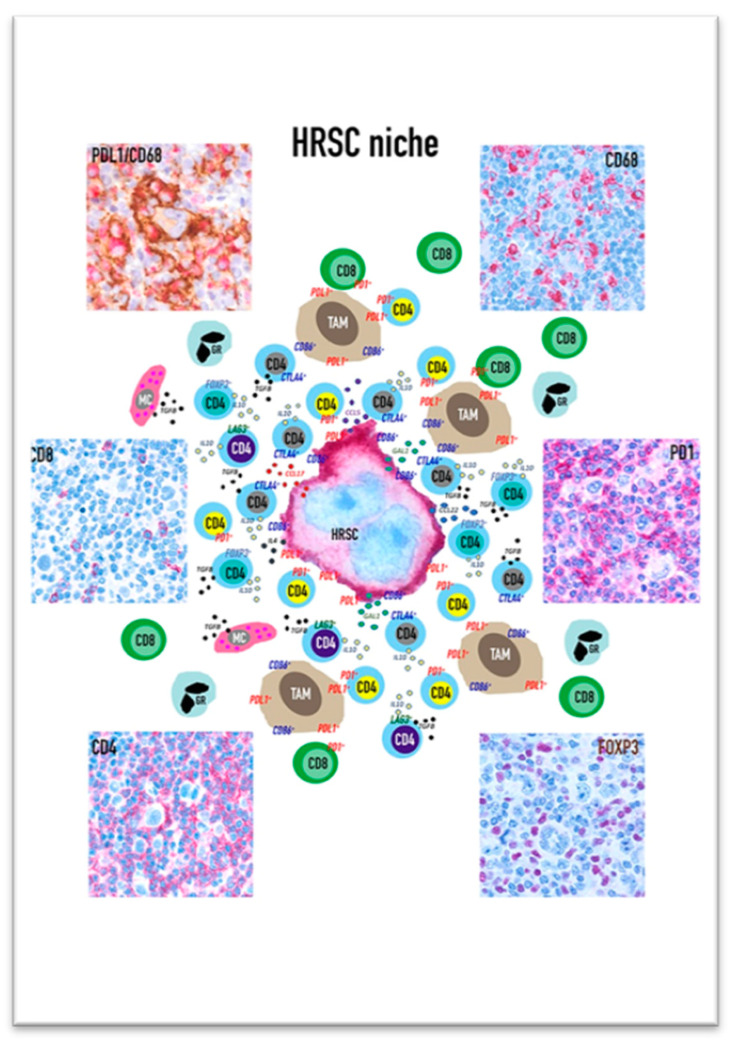
Representation of the Hodgkinian niche with CD30+ HRSC surrounded by several subsets of Th-1 and T-reg CD4+ lymphocytes, CD8+ cytotoxic T-cell, tumour associated macrophages (TAMs), granulocytes (GRs) and mast cells (MCs).

HRSCs play a major role in the organization of the ME milieu: they are able to reshape not only the functional status of immune populations but also the spatial distribution of the different cell subsets, creating a specialized biological niche that provides prosurvival signals to HRSC and shelters them from the antitumour immune response (Figure 1).

They can directly recruit several immune cell types from the peripheral circulation and trigger the local expansion of these cellular subsets and this recruitment of immune cells is also stimulated by reactive macrophages and mast cells. Specifically, HRS cells synthesize and release many cytokines and chemokines such as IL-5, IL-7, IL-8, IL-9, CCL-5, CCL-17, CCL-20 and CCL-22 that are involved in the recruitment of granulocytes, lymphocyte, mast cells and macrophages [16,17]. HRSC also express a broad range of receptors including CD30, CD40, IL-7R, IL-9R, IL-13R, TACI and CCR5 that are able to detect growth and survival signals coming from the background with the final effect of the delivery of prosurvival feedback to HRSC. 

As previously stated, the effects on the spatial distribution of microenvironmental components represents a consequence of specific recruitment by HRSC and at the same times allows the progress of interactions themselves.

In fact, although cHL neoplastic cells diffusely express PD-L1 (due to the presence of gain/amplification of locus 9p24.1), most of the tissue PD-L1 is localized on the cell surface of CD68+/CD163+ tumour associated macrophages (TAMs). PD-L1^+^ TAMs however are not spatially distributed in a random way, but they are significantly more abundant in the proximity of HRSC [18]. The expression of PD-L1 is induced by cytokines as INFγ or GM-CSF secreted by both HRSC and ME cells; interestingly a recent study demonstrated that PD-L1/L2 protein levels rapidly increase on monocytes via trogocytosis. The latter is an intercellular transfer of membrane patches from HRSC to monocytes in cHL and was not observed in monocytes cocultured with PD-L1/L2-deficient HRSC [19]. Since trogocytosis implies a cellular contact, this process could explain the enrichment of PD-L1+/PD-L2+ TAM close to HRSC and may further contribute to antitumour immunity evasion. Additionally, the several T cell subsets (Th-1, Th-2, Th-17, T-regs and cytotoxic lymphocytes) are not randomly distributed in the cHL ME. The CD4+ and CD8+ T cells including the PD-1+ subset are more numerous around and in contact with PDL-1+ TAM than PD-L1- TAMs suggesting that the PD-L1+ TAMs may either promote antitumour immunity through antigen presentation or immunosuppression through the engagement of PD-1 [18]. Moreover, CD4+ T lymphocytes, including PD-1+ ones, are enriched in proximity to HRSCs where they form rosettes in a fraction of cHL samples [18]. The low number of CD8+ T lymphocytes in the vicinity of or in contact with HRSC could be related to the frequent decrease or loss of β2M/MHC class I complex expression related to the inactivating mutations in β2M gene; conversely the CD4 richness around/in contact with HRSC may also be partly linked to the more frequent expression of MHC class II by the tumour cells. Recent studies have identified a high concentration of CD4+/CTLA-4+ T cells in the ME. Although CTLA-4 selectively identifies FOXP3+ regulatory cells, in cHL the CTLA-4+/FOXP3- T cells exceed the CTLA-4+/FOXP3+ ones and are highly enriched in proximity or in contact to HRSCs and to TAM. The CTLA-4+/FOXP3- cells are mostly PD-1- and engage the CD86, a cognate ligand for CTLA-4, which is expressed by HRSC and TAM. The expression of MHC class II can determine the T-reg cell types in the ME: the tissue of MHC-II positive cHL shows abundant CD4+/FOXP3+ T cells resetting the HRSCs, whereas in MCH class II negative cases the FOXP3+ T-regs are rare and HRSC are closely surrounded by CD4+/LAG-3+ T cells. As mentioned above, the latter are also CTLA-4+/CD25+ but negative for PD-1 and for the canonical T-reg marker FOXP3 suggesting they may exhibit a type 1 regulatory (Tr1) T-cell phenotype [20]. 

This biological niche not only allows an immune escape but transmits antiapoptotic and proproliferative stimuli to HRSC. The immune infiltrate, either through the release of cytokines that activate JAK/STAT signalling or by contact with the numerous surface receptors of HRSC, fuels the activation of NF-kB, PI3K/AKT and MAPK/ERK pathways. HRSCs express several tumour necrosis factor receptors: the CD40 ligand on CD4 T lymphocytes engages the CD40 on HRSC triggering the NF-κB pathway, CD30 binds the CD30 ligand on mast cells and eosinophils activating the PI3/AKT pathway, TACI and BCMA link BAFF and APRIL respectively on myeloid cells and, by recruiting TNF receptor-associated factor (TRAF) adaptor molecules, sustain NF-κB activation and upregulate Bcl-2, Bcl-xL and c-Myc. Moreover, HRSCs aberrantly express several receptor tyrosine kinases, despite the absence of activating mutations, such as DDR1, DDR2, EPHB1, TRKB, TRKA, c-MET and the MET-homologous receptor RON; the latter can be activated in a paracrine and autocrine fashions favouring the survival of the neoplastic clone [21,22,23]. 

Noteworthy, however, is the fact that the composition of the Hodgkinian niche is not uniform in all cHL samples, but it can be influenced by additional factors such as the presence of EBV or the expression pattern of MHC complexes. EBV-related cHL has a cytokine immune milieu dissimilar to that in EBV-cases, as it was associated to a high cytotoxic environment. In fact, EBV-related cHL shows an increased infiltration of CD8+ T cells associated with the expression of the Th1 transcription factor Tbet and Interferon gamma, displaying the profile of typical effective antitumour immunity. On the other hand, EBV− tumours manifested a Th17 profile and engagement of the IL-23/IL-17 axis with autoimmune and pro-oncogenic effects and the overexpression of GITR (TNF family member), which acts as a stimulator for T-regs cells [24,25].

Even if this finding sounds promising in the adult EBV-related cHL, some differences were detected in paediatric EBV positive cases where this effective response is counterbalanced by a microenvironmental niche enriched in PD-L1+ cells; underlining the age-related features of the immune system.

These findings suggest that EBV triggers a Th-1 mediated immune response directed against viral or tumour antigens that however might be counterbalanced by a microenvironmental niche enriched in PD-L1+ cells [26,27,28,29]. 

## 3. T Lymphocytes

T lymphocytes are the most represented component of cHL ME. The infiltrate is made up of both CD8+ and CD4+ T cells, the latter constituting often the largest population. At the site of disease, the recruited T lymphocytes surround HRSC and may form structures called “rosettes”. In the late 70s such structures were interpreted as an ineffective attempt to eliminate HRSC [30,31]. The inflammatory background contains abundant T helper (Th)-2 and IL-10 secreting T regulatory (T-reg) cells that are involved in the development and maintenance of an immunosuppressive ME [32,33]. In fact, HRSCs produce Th-2 and T-reg chemoattractants, including CCL17/TARC, CCL22, CCL5, IL-4, IL-5, IL-10 and IL-13 and induce a functional reprogramming of tumour-infiltrating T cells skewing CD4+ differentiation toward Th-2 and T-reg phenotypes [34,35]. In vitro experiments showed HRSC and HC selectively overexpress the immunoregulatory glycan-binding protein, galectin-1 (Gal1), through an AP1-dependent enhancer, and TGF-β: both proteins favour the secretion of Th-2 cytokines and the expansion of CD4+/CD25high/FOXP3+ T-reg cells [36]. However, several studies partially modified this picture of Hodgkin lymphoma ME demonstrating that Th-1/activation markers rather than Th-2/immunosuppression markers in T lymphocytes may play a major role. Graves et al. showed that T-lymphocytes express Th-1 associated CXCR3/CCR5 molecules and retain the capacity of TNFα/IFNγ/IL-2 cytokines secretion [37]. By applying mass cytometry to cell suspension from cHL biopsies, Cader and collaborators also identified selective expansion of more differentiated CD4+ Th-1 polarized T effector memory cells and Th-1 polarized T-regs in cHL cell suspensions [38]. New lymphocyte subpopulations have recently been identified in Hodgkin ME. Aoki et al. performed high-dimensional and spatial profiling of immune cells in cHL using scRNA-seq, multicolour immunohistochemistry and imaging mass cytometry identifying a unique regulatory CD4+/FOXP3+ T cell-like subset that expressed lymphocyte activation gene 3 (LAG3). These LAG3+ T cells demonstrated an immunosuppressive function mediated by the expression of IL-10 and TGF-β [20]. Moreover Ferrarini et al. found that HRS and surrounding T lymphocytes stained positive for IL-17 in 40% of cases and that the coculture of cHL cell lines with peripheral blood mononuclear cells promoted the enrichment of Th17 lymphocytes and FOXP3+/IL-17+ cells: these observations suggest the existence of an IL-17 tumour-shaped inflammatory milieu, at least in the subset of cHL cases [39]. At last, Le et al. described a previously unrecognized subset of CD8+ T cells exhibiting phenotypic and functional similarities with CD4+ follicular Th (Tfh) cells in a subgroup of patients. Similar to Tfh cells these CD8+ lymphocytes coexpress CXCR5, the inducible T-cell costimulator (ICOS) molecule, BCL6, PD-1, BTLA, CD200 and OX40 and downregulate CCR-7. They have deficient cytotoxicity, low INFγ secretion ability and functional properties in common with intratumoral CD4+ Tfh cells, such as the production of IL-4, IL-21, CXCL13 and capability to sustain B cells [40]. 

## 4. Macrophages

The finding that tumour associated macrophages (TAMs) and their interaction with neoplastic cells impacted on Hodgkin lymphoma prognosis dates back to 1985, when Ree and Kadin, described the immunohistochemical expression pattern of the peanut agglutinin (PNA). They concluded that a high number of macrophages was an important determinant in the clinical presentation and course of disease: the higher the number the worse the prognosis [41]. However, to date, despite several studies, the role of TAM in cHL has not yet been fully clarified. Macrophages are versatile cells characterized by an extraordinary functional plasticity: they can be immune-stimulatory or immune-suppressive, pro- or anti inflammation and in a tumoral setting they can favour or restrain disease development [42]. This heterogeneity has been oversimplified by the concept of functional polarization, which identifies two types of macrophages, M1 and M2, with distinct and opposite functions, induced by different types of cytokines present in the ME [43]. M1, or classically activated macrophages, are activated by Th-1 cytokines like INFγ and bacterial products; they secrete immunostimulatory cytokines that fuel the adaptive immune response and may acquire cytotoxic activity against transformed cells. M2, or alternatively activated macrophages, develop in a Th-2 cytokine-rich ME such as IL-4 and IL-13; they have high scavenging activity and produce several growth factors that activate the process of tissue repair and suppress adaptive immune responses [44,45]. The M2-like phenotype is often associated to protumour functions: in fact, these cells produce and release cytokines (IL-6) and growth factors (EGF, FGF, VEGF, PDGF and TGF-β), which stimulate proliferation and increase resistance to the apoptosis of tumour cells, activate fibroblasts and trigger angiogenesis. Furthermore, TAMs are the source of proteolytic enzymes that remodel the extracellular matrix favouring tissue invasion and metastasis [44]. In cHL, HRSC and HC recruit monocytes from the peripheral blood and can induce a M2 phenotype by the secretion of TNF, IL-10, TGF-β, GM-CSF, IL-13 and CCR5 [46,47]. The M2 macrophages may suppress cytotoxic T cell activity and attract T-reg lymphocytes, thus facilitating tumour growth and immune escape. In 2010 Steidl C et al. recognized a gene expression profiling signature of TAMs being associated with poor prognosis and correlated an increased number of CD68+ macrophages (as defined by immunohistochemistry) with a shorter PFS and a higher risk of relapse after autologous hematopoietic stem-cell transplantation [46]. Indeed, the prognostic value of the immunohistochemical quantification of TAMs exceeded IPS and was validated in the setting of multicentre phase III randomized controlled clinical trial E2496 [47]. However, although several studies confirmed that a higher density of TAMs is associated with inferior outcomes after first line treatment [48,49,50,51,52,53], others denied their negative impact in ABVD treated cHL. A possible explanation of these conflicting results can be ascribed to standardization of scoring methodologies. Jachimowicz et al. applied a whole-slide image analysis (WSI) that allows a quantitative, large-scale assessment of tumour composition utilizing conventional histopathology slides. They applied this approach to advanced-stage cHL samples from patients treated with BEACOPP-based regimens in the HD12, HD15 and HD18 trials of the German Hodgkin Study Group [54] and did not observe a correlation of macrophage content with outcome despite the cohort being enriched in vents [55]. A further issue might be the limits in the characterization of polarized macrophages in situ. CD163 was proposed as the marker of the M2-like phenotype and CD163+ TAMs and it indeed resulted in being a strong predictor of adverse outcomes in numerous series of ABVD treated cHL [48]; however, several authors challenge that CD163 can be considered a reliable M2 marker when used on its own. Barros et al. proposed a double-immunohistochemical labelling approach based on the detection of macrophage markers together with transcription factors regulating macrophage polarization, such as pSTAT1 and RBP-J for M1 and CMAF for M2 [21]. They demonstrated that predominant M1-like polarization, disclosed by the CD163+pSTAT1+/CD163+CMAF+ ratio > 1.5, was associated with better OS [21]. Since c-Myc was demonstrated to induce M2-like polarization by regulating genes associated with the alternative activation of macrophages, including SCARB1, ALOX15, MRC1 and CD209 [56], Werner et al. combined a macrophage-specific antibody (CD68 or CD163) to a reagent detecting c-MYC protein: they showed that MYC− macrophages (M1) are significantly higher in EBV+ cases while no differences exist in MYC+ macrophages (M2) between the EBV+ and EBV− cases [57]. Interestingly, they observed that a relative lack of TAM may allow cHL growth, intermediate numbers of TAM display an inhibitory effect and above a certain higher threshold, TAM may again support tumour growth [57]. These latter findings suggest that TAMs may have an hormetic rather than a linear relationship to outcome parameters and that their effects on survival may reflect underlying changes in macrophage polarization [57]. The mechanisms by which HRSC and HC modulate the TAM differentiation into alternatively activated tumour-promoting M2-like phenotypes are still not well defined. Functional studies showed that the global mRNA expression and protein quantity of macrophages in the cHL-conditioned medium differ from those of macrophages exposed to conditioned media from different diseases such as diffuse large B-cell lymphoma (DLBCL) cells or by a macrophage colony-stimulating factor (M-CSF). Conditioned medium from cHL cells triggers TAM differentiation into a M2-like phenotype via JAK/STAT signalling activation by the upregulation of surface antigens such as CD40, CD163, CD206 and PDL1 [58]. In particular, high mannose receptor C type 1 (MRC1 or CD206) enhances mannose-dependent endocytosis and type I collagen uptake contributing to matrix-remodelling and preclinical models revealed that cocultures of cHL cells with monocytes/macrophages support the dissemination of lymphoma cells via lymphatic vessels [58]. Finally, CD206 high IHC expression displays a positive correlation with an advanced stage of HL patients [58]. Given that M2-like TAM likely favour cHL progression, the possibility of reconverting the macrophage phenotype towards a proinflammatory M1 status could be an intriguing therapeutic perspective. An anticipation of this possible scenario comes from trabectedin (ET-743, Yondelis). Trabectedin is an alkylating agent that binds to DNA at the N2 position of guanine promoting degradation of RNA polymerase and generating DNA double-strand breaks, blocking the cell cycle and decreasing cytokines production. This anticancer drug is already approved as second-line therapy for ovarian cancer and soft tissue sarcomas. Its immunomodulating activities have been also demonstrated in preclinical models of hematologic malignancies. Casagrande et al. recently demonstrated that it overcomes doxorubicin-resistance and decreases xenograft growth in cHL [59]. Moreover, conditioned medium from trabectedin-treated HRSC showed less chemoattractivity toward monocytes, which also expressed lower levels of indoleamine 2,3-dioxygenase-1, CD206 and PD-L1, and showed less production of IL-10, TARC and TGF-β. This lead to less inhibition of activated lymphocyte growth [59]. Promising results are also coming from the usage of the PI3Kδ/γ inhibitor RP6530 both in preclinical and clinical models [60]. Since recent data in solid tumours show that the selective targeting of the γ isoform of PI3K in TAMs modulates the immunosuppressive tissue ME resulting in tumour regression, Locatelli et al. investigated the activity of RP6530 in vitro and in vivo in cHL cell line xenografts [60]. They demonstrated that in vitro RP6530 not only had a direct cytotoxic effect on Hodgkin lymphoma cells, switching the activation of macrophages from an immunosuppressive M2-like phenotype to a more inflammatory M1-like state, through the downregulation of lactic acid metabolism. In addition, in human tumour xenografts, RP6530 repolarized TAMs into proinflammatory macrophages inhibiting neoangiogenesis, leading to tumour regression [60]. Furthermore, patients enrolled in a phase I trial using RP6530 that achieved a complete and partial response and showed a significant inhibition of circulating myeloid-derived suppressor cells (MDSCs) and an average mean reduction in the serum TARC level [60]. These data represent the first evidence of therapeutic-induced suppression of the malignant cell growth and ME reshape in CHL.

## 5. B-Lymphocytes and Plasma Cells

The role of B cells in cHL is significantly less clear and defined and the insights we have derive more from the clinical scenario than from specific biological assays. Non-malignant B cells are prevalent in nodular lymphocyte predominant HL, which can be successfully treated with anti-CD20 monoclonal antibodies [61], however, they are also present in the ME of cHL and targeting B cells with rituximab both as a single agent and in combination with a standard scheme as ABVD could bring benefits, particularly on B symptoms and in the advanced stage of the neoplasm [62,63,64]. Several studies utilizing gene expression profiling and/or immunohistochemistry suggested a favourable effect for reactive B cells in cHL. Greaves et al. demonstrated that a high non-follicular CD20+ cell density confers a superior 5-year OS in cHL patients but not a longer FFTF (freedom from first-line treatment failure) and disease specific survival [37]. In line with this latter finding Panico et al. reported that high CD20+ background cells predicted a favourable outcome in cHL and antagonized CD68+ macrophages, while depletion of CD20+ cells together with an increase of TAM identifies a group of patients with high-risk disease [65]. Chetaille et al. profiled cHL samples, using DNA microarrays, and observed that cases with a favourable outcome overexpressed genes specific for B cells [66] and recently Jachimowicz et al. found that low B cell count was associated with a significantly reduced PFS and OS in cHL patients treated with BEACOPP-based regimens [54]. These studies suggest an antitumoral role for B cell in cHL and indicate that they could be central actors rather than bystanders in the local immune reaction. However, existing data on the role of tumour-associated B cells (TAB) indicate a tumour promoting function in solid tumour where their immunoinhibitory function resembles that of regulatory B cells (B-regs), which increase in tumour progression and are a proven source of inhibitory cytokines such as IL-10 and TGF-β [67]. Additionally, in mouse cancer models, TABs are required for establishing a chronic inflammatory state that promote de novo carcinogenesis [68] and are able to orchestrate and sustain melanoma inflammation. In this setting, TABs represent a predictor for survival and response to immune checkpoint blockade therapy [69]. Increased IL-10+ B cell numbers in ME can also be associated by increased numbers of Foxp3+ Tregs, which are independently associated with tumour progression or reduced patient survival [70].

Whether or not plasma cells (PCs) play a role in the dynamics of the ME in cHL has not yet been the subject of in-depth research. Their clinical relevance diverges in the tumour ME of different malignancies: infiltration of PC is associated with poor prognosis in colorectal cancer, breast cancer and ovarian cancer but it is associated with a favourable outcome in the ME of non-small lung cancer [71,72,73,74]. Gholiha et al. investigated diagnostic tumour biopsies from patients with cHL and found that CD138+ PC were associated with the presence of B-symptoms, advanced stage and eosinophil infiltration [75]. Moreover, patients with high proportion of PCs (defined as ≥10%) have an inferior event free survival (EFS) and OS although significance was not maintained in the multivariate analysis [76]. PC and eosinophils are attracted by CCL28, produced by HRSC and HC and these cells also secrete interleukin-6 and IL-21, involved in plasma cell differentiation. Interestingly, Thompson et al. correlated elevated quantitates of serum free immunoglobulin kappa and lambda light chains (free light chain: FLC), present in 30% of CHL patients, with inferior EFS and OS [76]. Being the HRSC and HC incapable of secreting functional Ig molecules the authors suggested that polyclonal FLC were secreted by the polyclonal PC of the ME. It was hypothesized that patients with elevated FLC have elevations in inflammatory cytokines and chemokines. Although these studies indicate that PC can adversely affect prognosis, the way they facilitate the survival of the neoplastic clone is still unknown. It was demonstrated that that IgA+ immunosuppressive PCs infiltrate therapy-resistant prostate cancer, where it induces CD8+ cell exhaustion and suppresses antitumour cytotoxic T lymphocyte responses through PD-L1 and IL-10 [77]. In squamous carcinoma during tumour development, autoantibody production by PCs leads to the deposition of immune complexes within neoplastic tissue. These later, through activating FcγR, trigger several protumour pathways, including angiogenic, tissue remodelling and prosurvival pathways and regulate the response to chemotherapy [78].

## 6. Mast Cells

cHL is frequently associated with mast cell (MC) infiltration and quantitative studies showed that the population of MC is significantly higher in nodular sclerosis (NS) than in other subtypes [79]. Several experimental data indicate that in NS-cHL, MCs promote fibrosis releasing TGF-β, which induces fibroblast proliferation, migration, contraction and collagen production. It has been hypothesized that IL-13 production by HRSC may lead to fibrosis and furthermore, promote MC proliferation and infiltration. the MC in turn might further produce the fibrotic cytokines IL-13 and TGF-β, resulting in fibrosis typical of NS-cHL [80]. Moreover, in immunodeficient NOD/SCID mice inoculated with both MC and human HRSC, a more marked fibrotic component, was detected compared to tumours in mice that have been inoculated with HRSC alone [81]. Interestingly, the fibrosis inducing tension and compression among cells was demonstrated to help cancer progression and tumour cell invasion through the increase the local concentration of growth factors and cytokines, one can assume that fibrosis induced by MC could also contribute to cHL progression [82]. Furthermore, MC can promote the differentiation of T cells into FOXP3+/CD25+ Treg cells via the secretion of TGF-β and may be the major source of pro-tumorigenic angiogenic and lymphangiogenic factors [83]. However, depending on the cytokine milieu of tumour ME, MC can conversely release antitumorigenic molecules, such as TNF-α and IL-9 [84]. The role of MC could therefore be cancer specific. Indeed, in thyroid, gastric, pancreatic and bladder cancers and in Merkel cell carcinomas, non-Hodgkin lymphomas and plasmacytoma, MC seems to be protumorigenic and associated with poor prognosis, while in others (e.g., breast cancer) they seem to have a protective role [85,86,87,88,89,90,91,92,93,94,95]. In cHL MC infiltration correlates with poor prognosis: Molin et al. reported a worse relapse-free survival in patients with higher mast cell density and Andersen et al. reported a significantly poorer outcome only in mixed cellularity CHL [96,97]. The latter also showed that the number of MC inversely correlated with the numbers of TAM and cytotoxic cells. Studies on the biological mechanisms underlying this adverse outcome are lacking: in the inflammatory background, MC were the predominant CD30L-positive cells and were able to activate HRSC in vitro through the CD30L–CD30 interaction. MC can however also promote the growth of Hodgkin’s tumours by modifying the tumour ME with the release of vascular endothelial growth factor-A and the induction of neovascularization [81,98]. There are increasing evidence supporting that targeting MC and/or their mediators represents a potential therapeutic target. Bortezomib inhibits degranulation and bortezomib-treated MC lose the ability to induce fibrosis and neoangiogenesis and are not able to promote the growth of the Hodgkin tumour in vivo [81]. Hansen et al. [98] showed that all Hodgkin cells release extracellular vesicles (EVs) containing CD30 (CD30EV) as a membrane protein. These EVs bind to CD30L on bystander cells and present additional membrane-associated CD30 sites for the binding of SGN-35. This study suggests that this mechanism allows a double targeting of both cancer and bystander cells along with MC and eosinophils.

## 7. The Myeloid Derived Suppressor Cells

The myeloid derived suppressor cells (MDSCs) are a heterogeneous population of immature myeloid cells produced by the bone marrow in neoplastic settings: when stimulated by chemical mediators (GM-CSF, G-CSF, M-CSF, VEGF, IL-1β, IL-4, Il-6, IL-10, IL-13 and IFNγ) they differentiate into monocytes and granulocytes, enter the bloodstream and reach the target organs where they perform an immunomodulatory function. In particular, they exert an immunosuppressive function both through the direct production of TGF-beta, ROS, IL-10 and peroxynitrite and through the indirect induction of T-reg differentiation in lymphocytes, thus promoting tumour growth and survival. In particular, MDSCs mostly exert the immune suppressive function against T-lymphocytes: this mostly derives from the high expression of arginase (Arg-1) that induces the depletion of tissue ME-arginine, which is an essential amino acid for the effective function of T-cell receptor (TCR) zeta chain assembly and downstream signalling. All this profoundly suppresses T cell immune responses [99,100,101,102,103]. The accumulation of MDSC has been reported in the ME of many cancer types where they can also differentiate into TAM and promote tumour angiogenesis and metastasis formation. In cHL tissue samples, a high number of Arg-1+ myeloid cells are significantly associated to shorter PFS in early stage patients and generally correlated with a worse OS [104]. However, the difficulty in defining the precise antigenic profile of MDSC, which overlaps with the granulocytic and monocytic phenotypes, hinders a thorough evaluation of their role in the disease. In the attempt to find a peripheral blood marker that could biologically mirror the dysregulated MDSC in tissue ME, Romano A et al. showed that the three main circulating MDSC derived subtypes (monocytic, granulocytic and CD34+ fractions) are increased in cHL and that CD34+ MDSCs predict short PFS, similarly to PET-2 [105]. The same group found that elevated serum Arg-1 (s-Arg-1) levels are associated with a shorter PFS and proposed the enzyme to be a novel potential biomarker for cHL prognosis [106]. Interestingly they also demonstrated that treatment with brentuximab vedotin reduced the absolute number of both the three MDSC subtypes and the s-Arg-1 levels [107]. Furthermore, patients with cHL experiencing objective responses (complete and partial responses) in a phase I trial using the PI3Kδ/γ inhibitor RP6530, managed to show a significant inhibition of circulating MDSC: these observations reinforce the possibility that MDSCs can serve as a therapeutic target [107].

## 8. Natural Killer Cells

Natural killer (NK) cells belong to group 1, innate lymphoid cells, and are a heterogeneous cell population with different functions and tissue distribution [108]. In peripheral blood two types of NK cells coexist: the CD56brightCD16– subset specialized in IFN secretion and the more cytotoxic subset CD56dimCD16+. NK cells are involved in tumour immune surveillance and recognize the transformed cells that downregulated MHC class I through their inhibitory receptors (KIRs) and NKG2x/CD94. NK cells can actually also eliminate cancers that retain MHC class I provided they express activating ligands, which engage activating receptors on NK cells (NKG2D, DNAM-1, NKp46, NKp30 and NKp44). Compromised NK cell activity was reported in many solid and haematological malignancies, which are able to alter NK cell maturation, to directly suppress their function, to enrich the ME of less cytotoxic CD56brightCD16– NK cells and to decrease or increase the levels of activating and inhibitory NK receptors, respectively. Tissue ME of cHL contains low levels of CD56-expressing NK cells and few studies indicated a dysregulation of NK function [109,110]. In particular, in the blood of cHL patients a new subset of mature NK cells was found lacking DNAM-1 expression, having limited killing activity and being poor INFγ producers [110]. These findings could have important clinical implications for the design of NK cell-targeting therapies.

## 9. HRCs Escape the Immunosurveillance

In cHL, the inflammatory ME often accounts for more than 90% of the lesion; HRSCs must therefore utilize multiple strategies to evade the immune surveillance. We previously highlighted their ability to reprogram immune populations toward functional phenotypes that are not effective in the antitumour response. However, HRSC can also reduce or lose their antigen presenting function making themselves invisible to the immune system and trigger exhaustion programs in the ME lymphocytes and NK cells. β2M inactivating mutations disrupt the expression of the β2-microglobulin (β2M)/MHC class I dual protein complex at the HRSC cell surface and consistently only 20% of the cHL cases show positive membrane immunohistochemical staining for β2M or MHC class I proteins. Patients with decreased/absent β2M/MHC class I have shorter PFS, underlining the importance of MHC class I mediated antigen presentation by HRSC to cytotoxic T cells for an optimal response to standard therapy [111]. In addition, decreased/absent protein expression of also MHC class II is observed in about 70% of the cases; in 15% of cHL cases this is a functional consequence of the rearrangement of the major histocompatibility complex (MHC) class II transactivator (CIITA), which is a master regulator of HLA class II transcription [111,112]. Homozygous deletions within the CD58 gene were reported in cHL and lead to the loss of protein expression in a subset of relapsed disease. CD58 is involved in the immune recognition of the tumour cells via binding of the CD2 receptor expressed by most cytotoxic CD8+ T cells and NK cells: its downregulation further represent an immune escape mechanism of HL tumour cells, at least in the clinically aggressive disease [113,114]. Beside perturbing the process of antigen presentation, HRSC are also skilled in triggering exhaustion programs in T cells. Activation of T cells is mediated by the interaction of the T-cell receptor (TCR) with an MHC-bound antigen and by costimulation of coreceptors on the surface of the antigen presenting cells (APCs) and the T cells. However, in order to prevent the tissue damage during the immune reaction, signals that limit TCR activation are simultaneously triggered by coinhibitory coreceptors called negative immune checkpoints (ICPs). Persistent stimulation of the latter induces T-cell functional exhaustion, characterized by a hyporesponsive state, a decreased production of effector cytokines, an inhibition of proliferation and a reduced cytotoxic activity. Binding of PD-1 by its ligands PD-L1/PD-L2 is the major coinhibitory pathway regulating T-cell exhaustion, which is nevertheless a reversible process. PD-1 can be upregulated on activated T cells, NK cells, B lymphocytes and macrophages and was reported to be variably present in cHL ME also in T cells rosetting around HRSCs. PD-L1 was found to be expressed by TAM, APCs and by neoplastic cells of several cancers and hematologic malignancies [115,116]. In cHL the copy gain or amplification of the 9p24.1 locus that include PDL1/PD-L2/JAK-2 genes results in the constitutive expression of PD-L1 and PD-L2 in more than 85% of the patients [117]. A genetic base for the enhanced PD-L1/PD-1 signalling is extremely rare among neoplasms and could at least partially explain the impressive overall response rate (about 70%) to the PD-1 blockade by monoclonal anti-PD1 antibodies (nivolumab and pembrolizumab, Table 1) observed in relapsed and refractory cHL patients [14,15]. More recently, the combination of nivolumab with AVD was tested in patients with newly diagnosed, advanced-stage disease and was found to be highly effective, with an overall response rate of 87% and a complete response rate of 67% [118]. Furthermore, several trials are also evaluating the association of BV and nivolumab, showing that this combination could be an active and well-tolerated first salvage regimen in relapsed or refractory cHL, and being also active in previously untreated older patients with comorbidities [119,120]. In cHL other corepressors capable of inducing functional exhaustion/anergy of T cells are however present. Cytotoxic T-lymphocyte antigen 4 (CTLA-4) is a member of the immunoglobulin superfamily: it is expressed on activated CD4+ or CD8+ lymphocytes and competes with its homologCD28 receptor for the engagement of their cognate ligands, CD80(B7.1)/CD86(B7.2) on APC. CTLA-4 regulates the development and functioning of T-reg and is a key negative regulator of the T-cell response. Hodgkin ME is enriched of both CD4+/CTLA-4+/FOXP3+ regulatory cells and CTLA-4+ non-regulatory T lymphocytes, whereas CD86 was found to be expressed in TAM and HRSC [115]. These findings suggest that the CTLA-4:CD86 axis may play a role in the creation of an immune-tolerant ME and that patients with cHL may also benefit from the CTLA-4 blockade. Clinical trials evaluating the effects of the administration of the anti-CTLA-4 antibody (ipilimumab) in combination with nivolumab or nivolumab and BV are ongoing [117,121]. The lymphocyte activation gene-3 (LAG-3) molecule is on the cell surface of activated CD4+ and CD8+ effector T cells, CD4+FOXP3+ Treg, Tr1 cells, B cells, plasmacytoid dendritic cells and NK cells. It structurally resembles the CD4 coreceptor and binds to MHC class II with high affinity [122,123]. LAG-3 acts synergistically with PD-1 and/or CTLA-4 to negatively regulate T cell expansion and LAG-3+ T cells populate cHL ME in most of the cases [115,124,125,126]. As mentioned above, a distinct CD4+/LAG-3+/FOXP3− T cell subset, expressing IL-10 and TGFβ, was recently identified by Aoki et al [20].; it probably corresponds to a Th1-type T-regs population, able to reduce both proliferation and TNF production in LAG-3− T cells and previously demonstrated to suppress effector CD8+ T-cell function in cHL [38]. A clinical study investigating the preliminary efficacy of the anti-LAG-3 monoclonal antibody BMS-986016 in combination with nivolumab in relapsed or refractory cHL is currently ongoing (clinicaltrials.gov identifier: 02061761). Immune checkpoint regulator T cell immunoglobulin-3 (TIM-3) is a type I transmembrane protein that is expressed on T-regs, NK cells, monocytes, macrophages and dendritic cells. TIM-3 plays an important role in the induction of T cell tolerance and TIM-3+ T-regs were correlated to an exhausted CD8+ T phenotype in tumour ME [126]. TIM-3 is nearly always expressed in the ME of cHL and may be targeted in the treatment of relapsed/refractory HL [120]. Moreover, T cell immunoglobulin and the ITIM domain (TIGIT) is a coinhibitory transmembrane glycoprotein expressed on CD8+ cytotoxic T cells, CD4+ Th cells and FOXP3+ T-regs, which has a critical role in regulating exhausted CD8+ T cell responses in tumours [127,128] Li and coworkers found a variable proportion of TIGIT+/PD-1+ in cHL samples and this combination represents an attractive candidate for coblockade with the PD-1 pathway inhibitor [128]. 

## 10. Conclusions

cHL is a unique neoplasm because it is largely composed of a non-neoplastic inflammatory infiltrate. Due to the production of a large number of cytokines, HC and HRSC attract and favour the expansion of different immune cell populations, modifying their functional status in order to receive prosurvival stimuli and to turn off the antitumour immune response. To this purpose HRSC shape a biological niche by organizing the spatial distribution of cells in the ME.

Areas surrounding HRSC are enriched in CD4+ T lymphocytes and PD-L1+/CD86+ macrophages. The interaction of negative immune checkpoints expressed on T cells with their respective ligands on macrophages and HRSC induces a state of local immune anergy. The prevalence of the CD4+ T lymphocyte subsets PD-1+, CTLA-4+ or LAG-3+ and the absence of MHC class I on HRSC suggest that the action of ICP inhibitors may be mediated by CD4+ T cells rather than by cytotoxic CD8 lymphocytes, as occurring in several solid neoplasms. Interestingly, the tumour-reactive cytotoxic CD4+ T cells were demonstrated to play a role in immunity against human cancers and several studies indicate that their activity may be enhanced by the ICP inhibitor treatment [129]. In line with these findings, circulating CD4+ cytotoxic T cells (CD4+/GrB+/PD-1+ Th-1 effector memory cells) are more abundant in patients with relapsed refractory cHL and expression of MHC class II, but not MHC class I, on HRSC was associated with the response to nivolumab in the CheckMate 205 trial. Recently, the effectiveness of the treatment with ICP inhibitors was related to the activation of innate effectors: the abundance of activated NK cells was associated with more favourable responses to the PD-1 blockade, which reverses the immune evasion mediated by the interaction of PD-1+ NK cells and PD-L1+monocytes/macrophages [130,131]. Moreover, Cader et al. identified a new CD3−/CD68+/CD4+/GrB+ subset that was associated with the response to PD-1 inhibitors in the blood and tissue ME of cHL patients; these cells may represent a monocyte population that utilize granzyme B to destroy antibody-coated targets via antibody-dependent cellular cytotoxicity [132]. As CD3−CD68+CD4+GrB+ elements also express IRF4, pSTAT1 and pS6, indicating a prior exposure to IF-γ, it was speculated that the CD4+ T cell might modulate innate cytotoxic effectors.

However, there is still much to discover about the microenvironment of cHL. Multistaining techniques, supported by image analysis systems and software, are useful in defining the phenotypic characteristics of immune subpopulations, but the structural and functional complexity of the tumour microenvironment will probably be further defined by the application of new technologies of digital spatial profiling. The latter being able to simultaneously detect, localize and quantify a large number of proteins and RNA molecules directly on tissue sections will allow one to further define the cellular composition and functional dynamics of the Hodgkin niche, providing a rationale for the appropriate use of ICPIs and new drugs that will target the microenvironment.

## Figures and Tables

**Table 1:** Immune checkpoint molecules.

Function	Name	Uses
**anti-PD1**	Nivolumab; Pembrolizumab	approved for metastatic melanoma, non-small cell lung cancer; refractory Hodgkin Lymphoma, advanced renal cell carcinoma, urothelial carcinoma
**anti-PDL1**	Atezolizumab, Durvalumab	approved for bladder cancer, non-small cell lung cancer, Merkel cell carcinoma
**anti-CTLA4**	Ipilimumab; Tremelimumab	approved for kin melanoma
**anti-Lag 3**	BMS986016	in study for cervical cancer, bladder cancer, colonic cancer, melanoma, noon small cell lung cancer

## Data Availability

Data sharing not applicable.
